# Cohort Profile: The 1934–66 Mysore Birth Records Cohort in South India

**DOI:** 10.1093/ije/dyv176

**Published:** 2015-10-07

**Authors:** Murali Krishna, Kumaran Kalyanaraman, SR Veena, GV Krishanveni, SC Karat, Vanessa Cox, Patsy Coakley, Kiran Nagaraj, Claudia Stein, BDR Paul, Martin Prince, Clive Osmond, Caroline HD Fall

**Affiliations:** ^1^ Early Career Research Fellow, Wellcome DBT India Alliance, India,; ^2^ Epidemiology Research Unit, CSI Holdsworth Memorial Hospital, Mysore, India,; ^3^ MRC Lifecourse Epidemiology Unit, University of Southampton, Southampton, UK,; ^4^ Division of Information, Evidence, Research and Innovation at WHO/Europe, Copenhagen, Denmark and; ^5^ Institute of Psychiatry, Kings College, London, UK

## Why was the cohort set up?

Coronary heart disease (CHD) and type 2 diabetes are common and major causes of morbidity and mortality in low- and middle-income countries, including India. ^1^ In the early 1990s, small size at birth was shown to be associated with an increased risk of CHD and some of its risk factors like hypertension, type 2 diabetes and dyslipidaemia in UK, European and US populations. ^2–4^ These associations were independent of adult lifestyle (including smoking, obesity and social class) and led to the Barker Hypothesis, which proposed that adult cardiometabolic disorders are ‘programmed’ *in utero* , and result from persisting changes in metabolism and organ structure that had occurred in response to fetal and early postnatal undernutrition. ^4^^,^^5^ Studies in the 1980s had recorded high rates of CHD in Indian populations living in the UK, which were largely unexplained by known risk factors. ^6^^,^^7^ However, relationships between size at birth and adult cardiometabolic disorders had not been examined in India, where fetal growth restriction and small size at birth are common. India has a high incidence of low birthweight (nearly 30%)—one of the highest in the world. ^8^

In 1991, one of the authors (CHDF) wrote to over 300 long-established hospitals in India, to ask if any had preserved old birth records going back 25 years or more. The author subsequently visited the 12 hospitals that replied affirmatively and welcomed collaboration, to assess the completeness and quality of the records and the feasibility of tracing people born in the hospital many decades earlier. CSI (Church of South India) Holdsworth Memorial Hospital (HMH) in the city of Mysore, southern India, had preserved its obstetric records in hard copy from 1934 to the present. The records were in good condition and were remarkably complete for birth measurements (weight, length and head circumference). Furthermore, the relative isolation of Mysore has ensured stability of the population, and a tracing exercise showed that it was possible to locate people born in HMH as far back as 1934 and match them accurately to their birth records.

**Figure 1.**
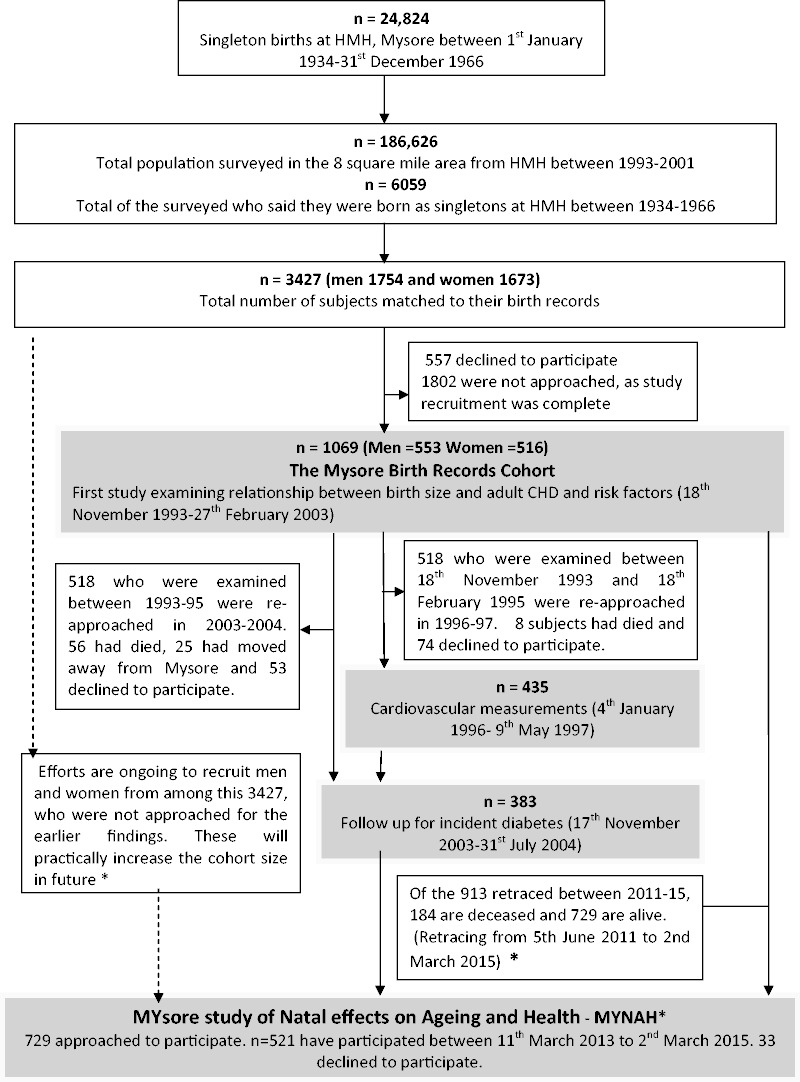
Mysore Birth Records Cohort. Asterisk designates ongoing tracing and recruitment for the present study.

Between 1993 and 2001, in a collaborative study with the Medical Research Council Environmental Epidemiology Unit (now Lifecourse Epidemiology Unit), University of Southampton, UK, the birth records at HMH were used to trace people born in the hospital between 1934 and 1966. A house-to-house survey, of an 8-square-mile section of the city surrounding HMH, located individuals who said they had been born in the hospital. They were matched to birth records using an algorithm; 3427 men and women born during 1934–66 were matched in this way and 1069 were recruited to the study (see Figure 1 ).

The first study ( *n* = 1069, 1993–2003) examined associations of size at birth with adult cardiometabolic disorders (type 2 diabetes, CHD, hypertension, insulin resistance, dyslipidaemia) and lung function. ^9–12^ Data were also collected on socioeconomic status, education, occupation, tobacco and alcohol consumption. Subsets of those who participated in the first study between 1993 and 1995 ( *n* = 518) were invited to participate in follow-up studies that examined:

- whether smaller size at birth was associated with alterations in the cardiovascular system that increased the risk for CHD (increased left ventricular mass and reduced arterial compliance), 2–3 years after the initial study, *n* = 435, 1996–97; ^13–15^
- whether smaller size at birth was associated with higher incident diabetes and CHD, 10 years after the initial study, *n* = 383, 2003–04; ^16^
- and a small intergenerational study between 1997 and 2003 examined associations between the birth size of members of the cohort and cardiometabolic risk factors of their children; this study included 506 adult offspring of cohort members. ^17^

## Who is in the cohort?

Holdsworth Memorial Hospital (HMH) is a mission hospital, now governed by the Church of South India. It was built as a maternity hospital in 1905 in a poor, crowded area of the city. At that time it was one of only two major maternity units in the city. Since 1934, birthweight, length and head circumference have been recorded routinely for all babies born in the hospital. Until the 1960s these measurements were made by one of three midwives, using an agreed protocol, although the details of how the measurements were made have been lost. The birth records also contain the parents’ names, occupations, address, religion or caste and the mother’s obstetric history. Some of the mothers attended the antenatal clinic, and their records (approximately 40%) include their weight during pregnancy, whereas others came to the hospital for the first time only when they were in labour. In the latter case, maternal weight was generally missing, but approximately 55% of mothers had pelvic diameters measured on admission in labour.

A total 24 824 singleton babies were born alive in HMH between 1934 and 1966. The house-to-house survey in 1993–2001 identified 6059 people who said they had been born as singletons in the hospital between 1934 and 1966, and of these 3427 were successfully matched to their birth records. The main difficulties in matching were that most adults were unaware of their date of birth and knew their age only vaguely, and that the birth records did not contain the infant’s name because Indian babies are usually named several weeks after birth. Cohort members were therefore matched to their birth record through their parents’ names, addresses and occupations, the number, sex and order of older siblings and the mother’s obstetric history. All of these details had to match exactly. Of the 3427, 1069 participated in the first study between 1993 and 2003 and constitute the Mysore Birth Records Cohort.

As a part of the current ongoing study we are recruiting men and women from the original 3427 matched to their birth records but who were not approached for the first study in 1993–2003. These will potentially boost the cohort numbers in future.

## How often has the cohort been followed up?

In 1996–97, 435 of the original cohort members, who had investigations between 1993 and 1995, were assessed for cardiac dimensions and arterial compliance.

In 2003–04, 383 of the original cohort members who had investigations between 1993 and 1995 participated in a follow-up study for incident diabetes and CHD.

There was no contact with the cohort between 2005 and 2011. The surviving members of this cohort are now being retraced and asked to participate in a follow-up study (MYNAH: MYsore study of Natal effects on Ageing and Health) to measure cognitive function, cardiometabolic disorders and mental disorders in late life. Figure 1 illustrates the numbers of participants in the past and current studies of the cohort .

The 3427 cohort members who were re-traced and matched to their birth records in 1993–2001 were only a small percentage (14%) of all the births in HMH during 1934–66, and of these approximately 30% ( *n* = 1069) took part in the initial study. Compared with the remainder of the original births, the 3427 individuals who were traced were 48 g heavier [95% confidence interval (CI) 28, 68, *p* < 0.001], 0.25 cm longer (95% CI 0.08, 0.42, *p* = 0.005) and had a 0.092-cm larger head circumference (95% CI −0.004, 0.188, *p* = 0.06) at birth. Compared with the 3427, the 1069 men and women who were studied were slightly lighter and shorter, but had similar head size [birthweight -36 g (95% CI −74, 2, *p* = 0.07); birth length −0.26 cm (95% CI −0.55, 0.03, *p* = 0.08); head circumference 0.07 cm (95% CI −0.09, 0.23, *p* = 0.39)].

When we attempted to re-trace a sub-sample of 518 in 2003–04 (10 years after the initial study) 56 (11%) had died, 25 (5%) had moved away from Mysore and we were unable to obtain any information about them and 53 (10%) did not wish to take part in a follow-up study. This made a loss to follow-up rate of 26%. The 383 men and women who were successfully re-studied were heavier and longer at birth compared with the remainder of the 517 [+75g (95% CI −3,154g, *p* = 0.06) and 0.62cm (95% CI 0.01,1.23, *p* = 0.045)] than those lost to follow-up. Looking at adult characteristics, they were of higher socioeconomic status [Kuppuswamy score +2.14 (95% CI 0.45, 3.84, *p* = 0.01)]. However, the groups were similar in age, gender mix, height, body mass index, educational attainment and prevalence of diabetes and clinical coronary heart disease in the earlier study.

Between 2011 and 2015 we attempted to re-trace the 1069 cohort members studied earlier, for a new study of the determinants of cognition and mental health in old age. Now a total of 184 (17%) have died and 156 (15%) could not be traced, leaving 729. Of these, 521 have participated in the ongoing study (until March 2015) and 33 have declined to participate.

Thus, as with all birth cohorts, especially cohorts of this age, there has been considerable attrition. Losses to follow-up will have occurred for a variety of reasons; some would have died in infancy and childhood, and this would have disproportionately affected smaller babies, which may explain the larger birth size in individuals who were traced in later life compared with the original births. Some would have moved out of the study area, and this is likely to have removed better-educated ‘upwardly mobile’ families, which may explain why the studied group was slightly smaller at birth than the non-studied group. Some individuals would have remained in the study area but could not be matched to their birth records due to incomplete information, and some adults would have been unaware that they were born in HMH. It is difficult to say how these various factors have influenced the representativeness of the traced cohort sample. The 10-year follow-up study suggested that men and women of higher birth size and socioeconomic status were more likely to survive, be traceable and/or agree to continue taking part in the research. Since the initial study in 1993–2003, 20% (184/913) of the traced cohort members have now died, and the cause of death was usually unknown (or unascertained). These losses could attenuate associations between newborn size and adult health outcomes among survivors but are unlikely to create spurious associations. In a within-cohort analysis, such bias would be introduced only if the associations between fetal growth and adult outcomes differed between those born in and outside the hospital, and in those traced and not traced.

## What has been measured?

### At birth

All 1069 participants in this cohort have contemporaneous data from their birth records, collected between 1934 and 1966 (data set 1, Box 1 ).

Box 1.Catalogue of the data collected from previous studies of the Mysore Birth Records Cohort. LDL, low-density lipoprotein; HDL, high-density lipoprotein

**Table:** 

*n = 3427*	Data set 1
*Matched birth records for cohort members born as singletons in HMH, Mysore between 1934 and 1966.*	Contemporaneous data from birth records: **Paternal** : occupation and religion. **Maternal** : age, weight (40%), years of marriage, parity, religion and pelvic measurements (55%) (interspinal, intercristal and external conjugate diameters). **Antenatal** : 40% of the records have date of last menstrual period, haemoglobin and blood pressure at antenatal visits. **Obstetric history** including birth order. **Labour data** : Maternal pulse and blood pressure; urine: specific gravity, sugar and albumin; course of labour, drugs administered and mode of delivery (normal, forceps or caesarean). **Child** : gender, birthweight, birth length and head circumference at birth. **Placenta** : insertion of cord, length of cord and cord condition. **Postpartum** : the day after delivery.
*n = 1069*	Data set 2
*First study of the cohort between 1993 and 2003. The participants were then aged between 40 and 60 years.*	**Blood tests:** Biochemistry: glucose tolerance test (WHO protocol, with blood taken fasting and 30 and 120 min after a 75-g glucose load), plasma insulin (fasting, 30 and 120 min post glucose), fibrinogen, factor VII, total cholesterol, triglycerides, LDL and HDL cholesterol, serum cortisol and cortisol-binding globulin ( *n* = 509). Haematology: full blood count, differential cell count, haemoglobin. **Anthropometry** : height, weight, head circumference, hip and waist circumferences and skin-fold thicknesses (triceps, biceps, subscapular, supra-iliac). **DNA** ( *n* = 551). **Clinical evaluation** : pulse rate, blood pressure, electrocardiographs (Minnesota coded), Rose angina questionnaire and spirometry ( *n* = 518). **Structured Interview:** Marital status, family structure and living arrangements. Medication, medical history of stroke, angina, hypertension, diabetes, chronic bronchitis and emphysema, history of coronary artery bypass graft or angioplasty. Family history of coronary heart disease and diabetes. d. Physical activity at home and at work Alcohol and tobacco consumption f. Dietary history: vegetarian/non-vegetarian, dietary intake of meat, dairy products, fish, fruits, vegetables and oils per week. **Kuppuswamy scale for socioeconomic indicators:** **^35^** locality in the town, household amenities (water, bathroom, toilet facilities), education level of subject and spouse, income of subject and spouse, occupation of subject and spouse, number of members in the household and number per room, total family income and per capita income. **Collected as a part of the tracing process** : birth order, family size, maternal and paternal occupation.
*After the initial study, a subset of the 1069 participated in follow-up studies; for these, in addition, to data set 1 and data set 2, the following additional data sets are available*	

*n = 435*	Data set 3
*Of the 1069, 435 examined between 1993 and 1995 participated in a follow-up study to examine cardiac dimensions and arterial compliance in 1996 –97. The participants were then aged between 43 and 63 years.*	Blood pressure, weight, height. Cardiac dimensions, pulse wave velocity, arterial compliance, left ventricular mass. Electrocardiograph. Hand Xray and osteoarthritis questionnaire. Reproductive history for women only (age of menarche, parity, pregnancies and breastfeeding). Medications
*n = 383*	Data set 4
*Of the 1069, 383 examined between 1993 and 1995 were followed up 10 years later between 2003 and 2004, to study incident diabetes. The participants were then aged between 50 and 70 years.*	**Blood tests:** Biochemistry: glucose tolerance test, plasma insulin (fasting, 30 and 120 min post glucose), fibrinogen, factor 7, total cholesterol, triglycerides, LDL and HDL cholesterol. Haematology: full blood count, differential cell count, haemoglobin. **Anthropometry** : height, weight, hip and waist circumferences and skin-fold thicknesses. **Clinical evaluation** : pulse rate, blood pressure, electrocardiography, spirometry, Rose angina questionnaire. **Structured Interview:** Marital status, family structure and living arrangements. Medications, medical history for stroke, angina, hypertension, diabetes, chronic bronchitis and emphysema, history of coronary artery bypass graft or angioplasty. Family history of CHD and diabetes. Physical activity at home and at work. Alcohol and tobacco consumption. Diet and food frequency schedule: vegetarian/non-vegetarian, dietary intake of meat, dairy products, fish, fruits, vegetables and oils per week. **Kuppuswamy scale for socioeconomic indicators:** **^35^** locality in the town, household amenities (water, bathroom and toilet facilities), education levels of subject and spouse, income of subject and spouse, occupation of subject and spouse, number of members in the household and number per room, total family income and per capita income. **Bio impedance recordings** : total body fat weight, lean weight, body water and metabolic rate. **Medical history** for peptic ulcer disease and *H. Pylori* infection and any surgical procedures.

### Between 40 and 70 yrs

Between 1993 and 2003, 1069 participants aged 40–60 years underwent assessment for anthropometry, CHD, abnormal glucose tolerance, dyslipidaemia and lung function (data set 2, Box 1 ). DNA material was collected from 551 participants. ^9–12^ 435 of those who were examined in the first study in 1993–95 returned for further cardiovascular investigations in 1996–97, aged 43–63 years. ^13–15^ Left ventricular mass was measured by 2D and M-mode echocardiography and blood pressure was recorded. Pulse wave velocity was measured by a non-invasive optical method using the principle of photoplesythmography, to estimate arterial compliance (data set 3 from Table1); 383 of those who were examined in the first study in 1993–95 were followed up at 10 years (between 2003 and 2004, aged 50–70 years) to assess incident diabetes and CHD (data set 4 from Box 1 ). ^16^ A blood sample for DNA was collected from 551 participants.

### Intergenerational study

An intergenerational study was carried out during 1997–2003. ^17^ Of 1069 members of the original cohort, 221 women and 166 men had adult sons and daughters also born at HMH. From the 221 mothers, we identified 206 sons and 209 daughters aged ≥ 20 years living in or near Mysore, making a total of 415 mother–offspring pairs. From the 166 fathers, we identified 152 sons and 144 daughters aged ≥ 20 years making a total of 296 father–offspring pairs. A total of 506 offspring (283 offspring of 193 mothers and 223 offspring of 144 fathers) aged 20–46 years participated in a study to examine the effect of parental size at birth and cardiometabolic risk factors in the adult offspring. They underwent assessments for anthropometry, CHD, abnormal glucose tolerance and dyslipidaemia. DNA material was collected from all the offspring who participated in the study (data set 2 from Box 1 ).

### Present study

We are presently recruiting surviving members of the cohort to a study examining the lifecourse predictors of cognition in late life. The investigations include:

- cognitive function as a continuous measure, obtained by administering a battery of cognitive tests developed, validated and normed in India by the 10/66 Dementia Research group; ^18–20^
- a structured clinical mental state interview (Geriatric Mental State), ^21^ an extended informant interview, ^19^^,^^20^ the History and Aetiology Schedule, ^22^ a structured neurological assessment (NEUROEX) ^23^^,^^24^ and the Neuropsychiatric Inventory to diagnose dementia and other mental disorders; ^25^
- assessments for chronic disease impairments, nutritional status, health behaviours and lifestyles, family living arrangements, economic status, social support and social networks; ^26–31^
- blood tests for diabetes, insulin resistance, dyslipidaemia, anaemia, vitamin B _12_ and folate deficiency, hyper-homocysteinemia, renal impairment and thyroid disease;
- anthropometry, ECG, Rose angina questionnaire, ^32^^,^^33^ blood pressure assessment, spirometry and a body composition analysis (bioimpedance);
- DNA sample for genetic assay of apoliprotein-E.

## What has it found? Key findings and publications

As in Western populations, among adults aged 40–65 years, lower birthweight, smaller head circumference and shorter body length at birth were associated with higher rates of adult CHD. ^9^ In addition, lower maternal weight during pregnancy was associated with a higher risk of CHD. This study provided early evidence from India that CHD is associated with fetal undernutrition. CHD was associated with some conventional risk factors including older age, shorter stature, diabetes, hypertension, altered concentrations of serum lipids, and smoking, but not with raised plasma fibrinogen concentrations or obesity. Prevalence rates were similar in men and women. There was a higher prevalence of the disease among men of lower social class, which was not explained by known risk factors. ^9^

Box 2.Key findings relating to coronary heart disease in Mysore Birth Records Cohort

- As in Western populations, among adults aged 40–65 years, lower birthweight, smaller head circumference, and shorter body length at birth were associated with higher rates of coronary heart disease (CHD).
- Lower maternal weight during pregnancy was associated with a higher risk of adult CHD.
- CHD was associated with some conventional risk factors including older age, shorter stature, diabetes, hypertension, altered concentrations of serum lipids, and smoking, but not with raised plasma fibrinogen concentrations or obesity.
- Prevalence rates of CHD were similar in men and women. There was a higher prevalence of the disease among men of lower social class, which was not explained by known risk factors.
- Associations between serum cortisol concentration and cardiovascular risk factors in this cohort were stronger than those previously shown in Caucasian populations, despite similar mean cortisol concentrations, and were amplified by adiposity.

The prevalence of type 2 diabetes was high (15%). Unlike studies in the West, however, it showed no association with lower birthweight. It was associated with shorter birth length, higher ponderal index at birth, higher maternal weight and larger maternal pelvic diameters. ^10^ These unexpected findings led to the hypothesis that maternal gestational diabetes, occurring among women who were stunted but obese, may be an important factor in causing high rates of type 2 diabetes in Indian urban populations. This led to the setting up of the Mysore Parthenon Study, a prospective cohort study of gestational diabetes. ^34^

Associations between serum cortisol concentration and cardiovascular risk factors in this cohort were stronger than those previously shown in Caucasian populations, despite similar mean cortisol concentrations, and were amplified by adiposity. ^11^ This suggested that increased glucocorticoid action may contribute to ethnic differences in the prevalence of the metabolic syndrome, particularly among men and women with a higher body mass index (BMI). Adult serum cortisol concentrations were not related to birth size.

Low birthweight and smaller head circumference at birth were associated with lower adult lung volumes, independent of gender and stature. ^12^ The effects of low birthweight and smoking were additive, so that the lowest lung volumes were found in smoking men who were small at birth. The association between low birthweight and reduced lung volumes in adult life is consistent with the hypothesis that fetal undernutrition has permanent effects on lung structure.

There were no associations between small size at birth and increased blood pressure and left ventricular mass, and reduced arterial compliance, in adult life. ^13–15^ At the 10-year follow-up, shorter birth length remained a risk factor for incident type 2 diabetes, whereas higher maternal weight and larger maternal pelvic diameters showed no association with incident disease. ^16^

The intergenerational study confirmed that factors in both parents were related to the risk of metabolic syndrome in their adult offspring. Both maternal and paternal birthweights were inversely related to metabolic syndrome in the offspring, independent of offspring age, sex, BMI and socioeconomic status. ^17^ These findings were consistent with the ‘fetal insulin’ (genetic) hypothesis. Maternal birthweight was inversely related to offspring systolic blood pressure. ^17^

## What are the main strengths and weaknesses?

### Strengths

This is the largest birth cohort in India in which surviving members are now in late life. There is good representation from both upper and lower socioeconomic classes and three major religions (Hindu, Muslim and Christian).

Detailed data on cardiometabolic disorders and risk factors, lifestyle and socioeconomic position in mid life are available for all, with a subset having cardiovascular measurements and repeat cardiometabolic measurements. Such data, spanning the lifecourse into old age, are hard to come by in low- and middle-income countries. This cohort therefore forms a unique resource for lifecourse studies examining cognitive, health and social outcomes in the elderly in a radically different cultural context from those in which such studies have previously been conducted.

The intergenerational data and DNA samples stored from the previous studies for the cohort members and their offspring forms an important resource for future studies examining the effects of genes and gene-environment interactions on size at birth, cardiometabolic risk factors and disorders, mental disorders and neurodegenerative disorders (like dementia) both within and across generations.

The ongoing study is the first lifecourse birth records cohort study in the developing world with cognitive, physical health and mental health outcomes in older adults. This will add to the scientific understanding of the early-life origins of disease and the mediating effect of cardiometabolic disorders in adult and late life on late-life cognitive abilities and mental health in an Indian population. In addition, this will establish a late-life baseline from which to explore pathways from early development to late-life impairment and decline.

### Weaknesses

The cohort is not population-based. It is based on all births within a single major hospital during selected years. The reputation of the hospital, which served the poor, was such that, many wealthy people chose to deliver there.

The protocol by which newborns were measured is unknown—for example, we do not know if birth length was measured using a standard stadiometer or using a simple measuring tape. Only 40% of the birth records include the date of the mother’s last menstrual period, allowing estimation of the gestational age. Similarly, only around 40% of the mothers had their weight or pelvic diameters recorded. Otherwise, there are no direct indices of maternal nutrition status for the cohort members. The birth records do not include placental weight. The newborns were not followed up in infancy or childhood, and so there are no measures of postnatal growth, which is also known to be related to adult disease and ageing.

As described and discussed in the section ‘How often has the cohort been followed up?’, there have been large losses to follow-up since birth, especially between birth and the first adult follow-up, and the implications of these losses have to be thought through in interpreting the results of individual studies.

## Can I get hold of the data? Where can I find more?

The study data are not freely available, but the Mysore Birth Records Cohort team would welcome collaborations with other researchers. For further information contact Dr SC Karat or Dr Murali Krishna based at Holdsworth Memorial Hospital, Mysore in India at [cshihmh@bsnl.in] or [muralidoc@gmail.com], or Prof. Caroline Fall at MRC Lifecourse Epidemiology Unit, Southampton in the UK at [chdf@mrc.soton.ac.uk].

Profile in a nutshell

- Between 1993 and 2001, 3427 men and women born as singletons during 1934–1966 at Holdsworth Memorial Hospital, Mysore, southern India, were traced by a house-to-house survey of the area of the city surrounding the hospital, and matched to their birth records. A total of 1069 of these participated in a study (1993–2003) to examine the relationship between size at birth and adult cardiometabolic disorders and lung function. They constitute the Mysore Birth Records Cohort.
- The studies with this cohort were among the first in a low- and middle-income country to test developmental origins of health and disease (DOHaD) concepts with predicted associations between small size at birth and adult coronary heart disease, insulin resistance and low lung function.
- This is one of few birth cohorts in a low- and middle-income country in which a substantial proportion of surviving members are aged over 60 years, and is a unique resource for lifecourse epidemiology studies in old age.
- Surviving members of this cohort are now being asked to participate in a cross-sectional study to measure cognitive function, cardiometabolic disorders and mental disorders in late life.
- Researchers wishing to access the data from this cohort should contact Dr SC Karat, Dr Murali Krishna or Prof. Caroline Fall.

## Funding

This work was funded by the: Medical Research Council, Parthenon Trust, Wessex Medical Trust, Wellcome Trust, Wellcome DBT India Alliance and Department for International Development, UK.

**Conflict of interest:** None of the authors report any conflict of interest.
